# Sphinkeeper Procedure for Treating Severe Faecal Incontinence—A Prospective Cohort Study

**DOI:** 10.3390/jcm10214965

**Published:** 2021-10-26

**Authors:** Christopher Dawoud, Leonhard Bender, Kerstin Melanie Widmann, Felix Harpain, Stefan Riss

**Affiliations:** Department of General Surgery, Division of Visceral Surgery, Medical University Vienna, 1090 Vienna, Austria; christopher.dawoud@meduniwien.ac.at (C.D.); n1326488@students.meduniwien.ac.at (L.B.); n01638813@students.meduniwien.ac.at (K.M.W.); felix.harpain@meduniwien.ac.at (F.H.)

**Keywords:** faecal incontinence, Sphinkeeper, sphincter lesion, artificial anal sphincter, endoanal ultrasound

## Abstract

(1) Background: The Sphinkeeper implantation for faecal incontinence (FI) is a novel surgical procedure with limited data on its clinical efficacy. Therefore, we aimed to assess the functional outcome following Sphinkeeper surgery in patients with refractory FI. (2) Methods: Between 2018 and 2020, eleven consecutive patients (9 female) with FI met the inclusion criteria and were enrolled for surgery. Functional outcome and quality of life were evaluated by standard questionnaires pre- and post-surgery. Migration of protheses was demonstrated by 3D endoanal ultrasound. The median follow-up time was eight months (range 3–18 months). (3) Results: The median age was 75 years (range 46–89 years) with a median BMI of 27.4 (range 21.2–30.1). The median number of implanted prostheses per intervention was nine (range 9–10). We found no intraoperative or early postoperative complications. After two months, two prostheses in one patient had to be removed due to pain at the perianal skin site. The median St. Mark’s incontinence score decreased significantly from 22 to 13 points (*p* = 0.008). The SF-12 showed a significant improvement (35.9 versus 46.3) after surgery (*p* = 0.028). A migration of at least one prosthesis was observed in ten patients (91%). Six (60%) prostheses were found at the same level in another ten patients. (4) Conclusion: Sphinkeeper implantation is a promising surgical technique for patients with severe FI. The complication rate is low, and short-term functional improvement can be achieved even in severe forms of FI. Migration of implants commonly occurs.

## 1. Introduction

Faecal incontinence (FI) is a multifactorial condition, which affects up to 20% of the general population [1,2,3]. FI significantly diminishes quality of life and can consequently also affect psychological well-being [2]. The management of FI can still be challenging. If conservative treatment fails, more invasive procedures, such as sacral neuromodulation, sphincter repair, or bulking agents are recommended. However, surgical options are still limited and new therapy approaches are needed.

In recent years, the Gatekeeper by THD SpA (Correggio, Italy) has been introduced in the treatment of FI [4]. In contrast to bulking agents, self-expandable solid prostheses made of inert Hyexpan (polyacrylonitrile) are inserted into the intersphincteric groove. The prostheses enlarge up to 700% of their original volume due to slow water absorption within 48 h after implantation. They are considered to cause constant pressure on the anal canal and thereby improve FI. It has also been suggested that an implanted prosthesis increases muscle fibre length, and therefore contributes to an enhanced contractility [5]. Few clinical studies are available, showing success rates in up to 50% of treated patients [6,7,8,9].

The initial Gatekeeper operation was then modified to the Sphinkeeper (SK) procedure (THD SpA, Correggio, Italy) in order to further increase functional outcome [6]. Hereby, ten modified prostheses, which are longer and larger than those of the Gatekeeper, were implanted using the same technique [6]. To date, literature on the Sphinkeeper is scarce; thus, its role in the management of FI is not yet well defined. Notably, a low number of short-term studies showed promising results with a significant decline in incontinence episodes [5,6,7,8,9].

The current study was designed to assess the overall pelvic floor function in patients with FI treated with the Sphinkeeper procedure.

## 2. Materials and Methods

A single-centre prospective observational study of patients treated with the Sphinkeeper procedure was conducted at the Medical University of Vienna. This study was performed in line with the principles of the Declaration of Helsinki. Approval was granted by the Ethics Committee of the Medical University of Vienna (#2338/2019).

Patients with FI, who failed to respond to conservative treatment, were eligible for inclusion. Written informed consent was required from all patients. Exclusion criteria were a malignant disease, rectal bleeding of unknown origin, and inflammatory bowel disease.

Notably, a sphincter defect (internus or externus) of more than 120° as well as chronic diarrhea unresponsive to medical treatment was not considered an exclusion criterion.

At baseline, demographic data (age, gender, body mass index [BMI]) and medical history with a particular focus on conditions affecting the anorectal function were recorded.

Functional outcome was assessed by using validated standardized questionnaires before surgery and at the last follow-up appointment. The St. Mark’s incontinence score was used to measure FI [10]. Obstructive defecation syndrome (ODS) and constipation were evaluated using the constipation score and the Rome III criteria [11,12]. Quality of life was measured by the Short-Form-Health survey (SF-12) [13].

At baseline and at follow-up, anorectal manometry (ARM) was conducted (THD Anopress, Pressprobe, Sensyprobe) to analyze resting, squeeze and straining pressure. The balloon test was used to define anorectal sensation.

Endoanal ultrasound (EAUS) (Flex Focus 500, BK Medical Holding Company, Inc., Denmark) was performed at baseline to evaluate anorectal morphology as well as anatomical anal canal length and after surgery to record the exact location of inserted prostheses during the follow-up.

### 2.1. Surgical Procedure

Each patient received an enema (Klistier Fressenius, 130 mL) before surgery. Antibiotic prophylaxis consisted of a single shot of 1.5 g metronidazole and 1.5 g cefuroxime intravenously.

The surgical procedure was performed with all patients in the lithotomy position, under general anaesthesia. Skin disinfection was performed with povidone-iodine solution. The procedure consisted of small perianal skin incisions (2 mm) made in about a two-centimeter distance to the anus. The introducer was inserted under EAUS guidance into the intersphincteric space. Additionally, before deploying the prothesis, the exact position of the tip of the introducer was controlled by using the EAUS. When the proximal segment of the prosthesis reaches the tip of the cannula, the entire cannula completely retracts inside the delivery system. Then, the prosthesis is released in the desired position of the intersphincteric space. Thereafter, the introducer was withdrawn and the next prosthesis was inserted in the same fashion with a 1 cm spacing, resulting in nine or ten implanted prosthesis. All operations were conducted by an experienced colorectal surgery consultant. After all prostheses were implanted, a final 3D EAUS was performed to record the final position of the implants. Skin wounds were closed using resorbable sutures. Patients were asked to remain in bed for 24 h to reduce the likelihood of prostheses migration.

### 2.2. Outcome Measurements

The primary outcome was defined as change of FI assessed by the St. Mark’s incontinence score. Secondary, the effect on constipation and QoL was assessed. Also, migration of the implants was recorded, and its impact on functional outcome was analyzed. We categorized the shifting of the prosthesis with their angle position to the anal canal through EAUS and divided them into three groups (0°–45°; 46°–90°; >90°).

### 2.3. Statistical Analysis

Continuous variables are expressed as median and range. Categorical variables are presented as numbers with percentages. To analyze primary and secondary endpoints, pre- and post-operative measurements were compared. Quantitative variables were compared using the dependent Student’s *t*-test, as appropriate. A *p*-value < 0.05 was considered to denote statistical significance. Statistical analysis was performed using the SPSS statistical software package (IBM SPSS Statistics for Mac, Version 22.0).

This work has been reported in line with the STROCSS criteria [14] and was performed using the STROBE checklist [15]

### 2.4. Study Registration

This study was registered at ClinicalTrials.gov (NCT04664868)—(Date of registration: 30 November 2020).

## 3. Results

### 3.1. Patient Characteristics

Between 2018 and 2020, eleven consecutive patients (9 female; 2 men), who met the inclusion criteria, were prospectively enrolled to this study and treated with a Sphinkeeper implantation. Demographic data are described in Table 1.

Six patients (55%) reported a vaginal delivery, whereas four patients (36%) had a perineal tear (grade 2–4). Seven patients (63%) had undergone previous surgery for FI, including sphincteroplasty, rectopexy and sacral nerve stimulation. Notably, five patients (45%) were complaining about simultaneous urinary incontinence.

The median duration of FI before surgery was 39 months (range 21–156).

### 3.2. Surgical Outcome

The median number of implanted prostheses was nine (range 9–10). The median operation time of EAUS-guided Sphinkeeper implantation was 44 min (range 34–53). We found no intraoperative or early postoperative complications during the initial hospitalization. The median hospital stay was two days (range 1–3).

**Figure 1:**
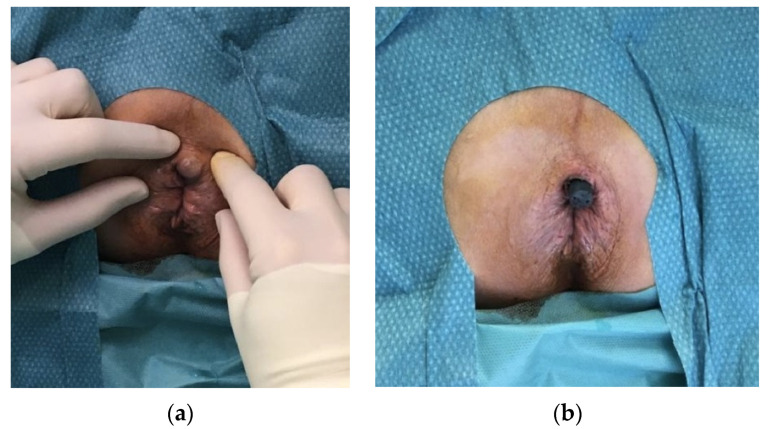
(**a**) Migration of a prosthesis towards the perineal skin. (**b**) Perineal skin already cut, with the prosthesis visible.

One patient complained about anal discomfort and mild pain after two months due to migration of two prostheses towards the perianal skin (see Figure 1a). The prostheses were removed without any complications and the pain was resolved (see Figure 1b).

### 3.3. Functional Outcome

**Figure 2:**
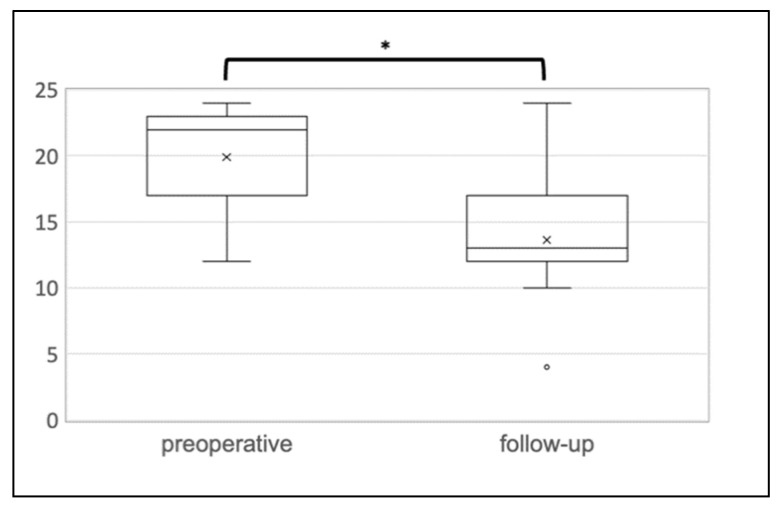
St. Mark’s incontinence score. The median St. Mark’s incontinence score decreased from 22 to 13 points (*p* = 0.008). * = significant; x = mean; ° = statistical outlier outside 1.5 times interquartile range.

**Figure 3:**
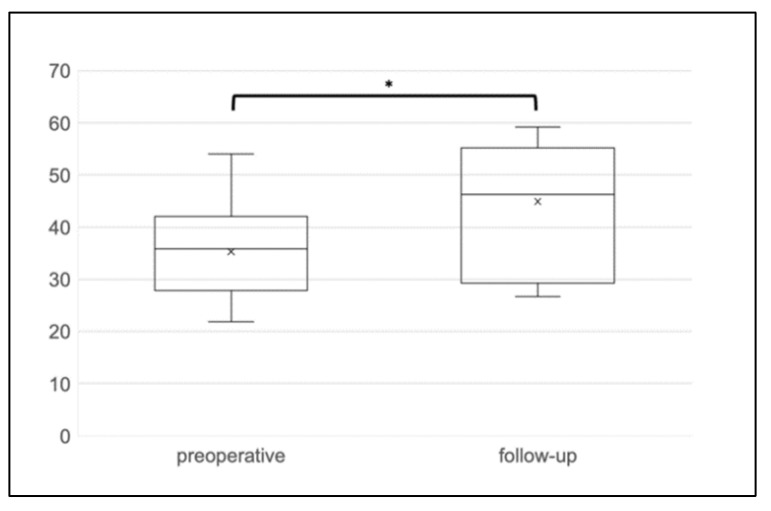
Short-Form-Health survey. The Short-Form-Health survey has increased in the follow-up significantly (35.9 vs. 46.3, *p* = 0.028). * = significant; x = mean.

The median follow-up time was eight months (range 3–18 months). The postoperative functional outcome is outlined in detail in Table 2. FI improved significantly following the Sphinkeeper procedure. The median St. Mark’s incontinence score decreased significantly from 22 to 13 points (*p* = 0.008) (see Figure 2). In contrast, the constipation score increased from 4.0 to 4.5 points without reaching statistical significance (*p* = 0.590). The Sphinkeeper procedure led to a considerable improvement of QoL as measured by the SF-12 (35.9 vs. 46.3, *p* = 0.028) (see Figure 3).

Resting, squeezing and straining pressure, as measured by anorectal manometry, did not differ from preoperative results. However, anorectal sensation has improved in most of the patients, but still lacking statistical significance (see Table 3).

The anatomical anal canal length has shortened significantly after implantation (*p* = 0.008).

### 3.4. Evaluation of Sphinkeeper Implants

**Figure 4:**
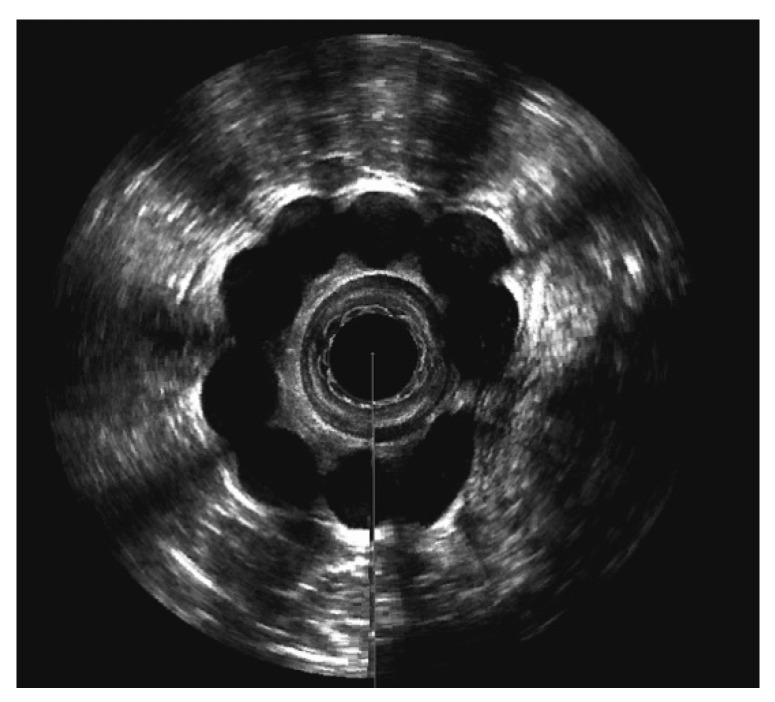
3D-endoanal ultrasound image of a patient at follow-up with nine Sphinkeeper prostheses.

All prosthesis reached their final size during our follow-up visits. In ten patients (91%), the prostheses were still placed in the intersphincteric groove at the last follow-up visit (see Figure 4). In one patient (9%), we found the prosthesis outside the external sphincter muscle. It is worth noting that this patient showed no improvement in faecal incontinence after the operation.

**Figure 5:**
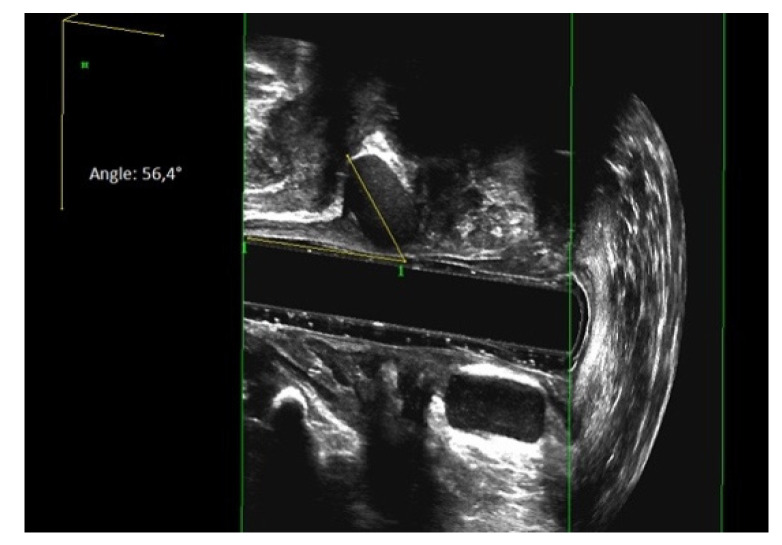
3D-endoanal ultrasound image: A migrated prosthesis in a 56.4° angle to the anal canal.

**Figure 6:**
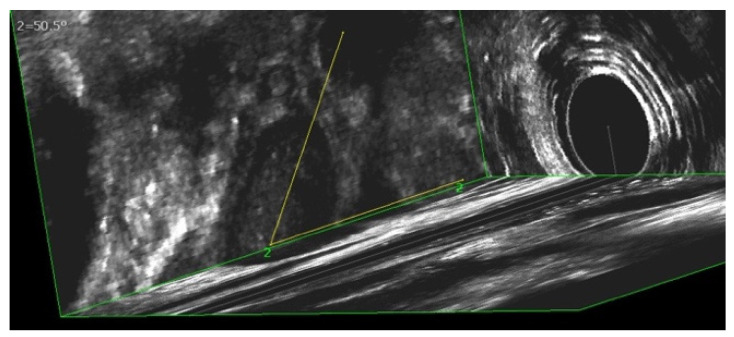
3D-endoanal ultrasound image: A migrated prosthesis in a 50.5° angle to the anal canal.

Nearly all patients (91%) showed a migration of at least one prosthesis. In ten patients, six (60%) prostheses were found at the same level. We categorized the position of the prosthesis in relation to the anal canal axis. The median number of prostheses remaining parallel (0°–45°) to the anal canal was six (range 5–9), while in median, two (range 0–4) prostheses showed a shifting grade I° (46°–90°). In median, zero prostheses (range 0–1) reached shifting grade II° (>90°) (see Figure 5 and Figure 6).

## 4. Discussion

Data on the functional efficacy of the novel Sphinkeeper procedure are still very limited. In addition, little is known about the migration tendency of inserted implants. In the present single-centre study, we revealed that patients reported a substantial improvement of their FI following Sphinkeeper surgery [10]. This was further accompanied by a better QoL. Interestingly, EAUS assessment could demonstrate a high rate of shifted implants. The rotation effect was seen in all patients, although the majority of prostheses remained in the intended position.

In general, Sphinkeeper surgery is safe, and complications rarely occur. This was also confirmed by other studies that found no serious adverse events during their follow-up periods [6,7,8,9]. It is worth noting that, in one patient, two implants migrated towards the perineal skin site causing discomfort during physical exercises, ultimately leading to the explantation of the prostheses.

To the authors’ knowledge, there are a paucity of data available on the functional outcome after Sphinkeeper surgery [6,7,8,9]. La Torre et al. included 13 patients and observed a lower number of FI episodes per week and a reduced Cleveland Clinical FI score after Sphinkeeper implantation [8]. Interestingly, anal manometry revealed a higher maximum resting pressure following surgery. In contrast to our study, indications for performing this procedure varied, as no patients with sphincter injury (IAS/EAS) of more than 70° were enrolled.

In line with our clinical findings, Leo et al. reported an improvement of the St. Mark’s incontinence score after Sphinkeeper implantation [9]. The authors describe that 51.9% of the patients achieved a 50% FI score reduction. The largest sample size to date was reported by Litta et al. and included 45 patients [7]. All types of incontinence decreased postoperatively. Five patients (11%) became fully continent [7].

The majority of available studies comprised patients with a St. Mark’s incontinence score between 14 and 15 points, reflecting a moderate form of incontinence. It is noteworthy that, in our series, most of the patients suffered from severe FI with a median score of 22 points. Thus, one could speculate that even severe levels of FI may improve with Sphinkeeper operation.

However, a certain number of patients did not respond to the procedure adequately. Unfortunately, patient selection parameters for this specific procedure still need to be defined.

Potential migration of the implants is an important aspect of the surgery. Current data are controversial concerning whether migration does have a negative impact on latter pelvic function. Ratto et al. included ten patients and found a dislocation of a single prosthesis in only one patient [6]. The patient became symptomatic and the pain was successfully treated with painkillers. Additionally, no migration, dislocation or extrusion of Sphinkeeper prostheses occurred. Trenti et al. studied the precursor, Gatekeeper, and found migration of prostheses in 51% of patients with a higher number of migrated prosthesis in the non-responder group [16].

Leo et al. showed a median of seven prostheses in follow-up in contrast to the implantation of nine at surgery [9]. Moreover, half of the visualized prostheses were seen in the right placement with no relationship between the number of well-sited prostheses and categorical success. The authors also reported that the firing device jammed and not all prostheses could be deployed, which did not happen in any of our case series [9]. It needs to be pointed out that the idea of the solid Sphinkeeper prostheses is its superior stability compared to the more common bulking agents. Several studies found that the effect of the bulking agents declines over time, which might be associated with the resorption of the bulking material [17].

The originally introduced Gatekeeper included a lower number of smaller prostheses [4]. Grossi et al. also compared the Gatekeeper to its successor Sphinkeeper [5]. They confirmed that both techniques offer the same benefit in terms of morphofunctional remodeling of the sphincter complex. Furthermore, they found an increased muscle tension in patients with a greater number of prostheses [5]. Whether the novel Sphinkeeper can further improve functional outcome is unclear and needs to be assessed in future studies.

Our study, like others, was limited by the inability to prove with certainty full surgical success. Another drawback of this study is the small sample and short follow-up period. However, it needs to be pointed out that we also enrolled patients with a more severe form of FI; thus, our data may help to further guide clinicians to select the appropriate patients. Because of the low number of recent studies, we still believe that the current series is of important clinical interest.

## 5. Conclusions

Sphinkeeper implantation is a promising surgical technique for patients with FI. It is safe and leads to short-term functional improvement, even in severe forms of FI. It has to be mentioned that not all patients may benefit from this procedure; thus, a selection criteria still needs to be defined. Migration of implants commonly occurs and its impact on the functional outcome remains unclear.

## Figures and Tables

**Table 1:** Demographics and baseline characteristics. Baseline characteristics of patients undergoing Sphinkeeper procedure. ASA: American Society of Anaesthesiologists score; BMI: body mass index; EAUS: Endoanal ultrasound; FI: faecal incontinence.

	*n* = 11
**Demographics**	
Age [years], median (range)	75 (46–89)
Female sex, *n* (%)	9 (81.8)
BMI [kg/m^2^], median (range)	27 (21–30)
**Clinical history, *n* (%)**	
ASA classification I/II/III/IV	0/6/5/0
History of smoking	3 (27.3)
Childbirth, *n* (%)	7 (77.8)
Perineal tear [grade], median (range)	0 (0–4)
Previous pelvic floor surgery, *n* (%)	7 (63.6)
sphincteroplasty surgery, *n* (%)	1 (9.1)
rectopexy, *n* (%)	4 (36.3)
sacral nerve stimulation, *n* (%)	5 (45.4)
EAUS—internal sphincter defect [grade], median (range)	67.5 (38–121)
**Stool parameters**	
FI form	
Active FI, *n* (%)	6 (54.5)
Passive FI, *n* (%)	2 (18.2)
Mixed FI, *n* (%)	3 (27.3)

**Table 2:** It shows the symptoms of faecal incontinence and obstructed defecation before and after operation in follow-up.

	Baseline *n* = 11	Follow-Up *n* = 11
**Faecal Incontinence symptoms**		
Incontinence to solid stool [daily episodes], *n* (%)	8 (72.7)	1 (9.1)
Incontinence to fluid stool [daily episodes], *n* (%)	8 (72.7)	1 (9.1)
Incontinence to gas [daily episodes], *n* (%)	7 (63.6)	2 (18.2)
Lifestyle alteration [daily], *n* (%)	8 (72.7)	2 (18.2)
Wears pad [daily], *n* (%)	11 (100)	8 (72.7)
**Obstructed defecation symptoms**		
Feeling of blockade in the rectum, *n* (%)	4 (36.4)	3 (27.3)
Feeling of incomplete defecation, *n* (%)	4 (36.4)	2 (18.2)
Strong squeezing in defecation, *n* (%)	2 (18.2)	3 (27.3)
Emptying effort >1 per day, *n* (%)	5 (45.5)	3 (27.2)

**Table 3:** It shows the different results of anorectal manometry and sensation before and after operation.

	Preoperative	Follow-Up	*p*
**Anorectal manometry**			
Resting pressure [mmHg], median (range)	15 (9–48)	16 (11–29)	0.593
Squeezing pressure [mmHg], median (range)	39 (33–90)	40 (21–85)	1.000
Straining pressure [mmHg], median (range)	19 (12–42)	34.5 (17–89)	0.109
**Anorectal sensation**			
first anorectal sensation [ml], median (range)	60 (10–60)	22.5 (10–60)	0.180
rectal tenesmus [ml], median (range)	140 (140–120)	45 (30–120)	0.109
maximal tolerated volume [ml], median (range)	220 (150–250)	70 (40–135)	0.109

## Data Availability

The raw datasets generated during and/or analyzed during the current study are not publicly available due to the sensitive nature of the questions asked in this study but are available from the corresponding author on reasonable request.
